# Influence of Environmental Drivers and Potential Interactions on the Distribution of Microbial Communities From Three Permanently Stratified Antarctic Lakes

**DOI:** 10.3389/fmicb.2019.01067

**Published:** 2019-05-15

**Authors:** Wei Li, Rachael M. Morgan-Kiss

**Affiliations:** ^1^Department of Land Resources and Environmental Sciences, Montana State University, Bozeman, MT, United States; ^2^Department of Microbiology, Miami University, Oxford, OH, United States

**Keywords:** aquatic protists, heterotrophic bacteria, interactions, environmental drivers, McMurdo Dry Valley lakes

## Abstract

The McMurdo Dry Valley (MDV) lakes represent unique habitats in the microbial world. Perennial ice covers protect liquid water columns from either significant allochthonous inputs or seasonal mixing, resulting in centuries of stable biogeochemistry. Extreme environmental conditions including low seasonal photosynthetically active radiation (PAR), near freezing temperatures, and oligotrophy have precluded higher trophic levels from the food webs. Despite these limitations, diverse microbial life flourishes in the stratified water columns, including Archaea, bacteria, fungi, protists, and viruses. While a few recent studies have applied next generation sequencing, a thorough understanding of the MDV lake microbial diversity and community structure is currently lacking. Here we used Illumina MiSeq sequencing of the 16S and 18S rRNA genes combined with a microscopic survey of key eukaryotes to compare the community structure and potential interactions among the bacterial and eukaryal communities within the water columns of Lakes Bonney (east and west lobes, ELB, and WLB, respectively) and Fryxell (FRX). Communities were distinct between the upper, oxic layers and the dark, anoxic waters, particularly among the bacterial communities residing in WLB and FRX. Both eukaryal and bacterial community structure was influenced by different biogeochemical parameters in the oxic and anoxic zones. Bacteria formed complex interaction networks which were lake-specific. Several eukaryotes exhibit potential interactions with bacteria in ELB and WLB, while interactions between these groups in the more productive FRX were relatively rare.

## Introduction

In aquatic ecosystems, the microbial loop plays a central role in fixation and transformation of energy and carbon, and the cycling of major nutrients. In addition, complex networks among the representatives of the microbial loop (viruses, Bacteria, Archaea, and microbial eukaryotes) have major implications on the global carbon cycle and other biogeochemical cycles (Azam et al., 1994; Azam, 1998; Cotner and Biddanda, 2002; Fenchel, 2008; Jiao et al., 2010). Investigating both microbial diversity and community structure is a crucial first step in understanding ecological functioning in aquatic environments. In many ecosystems, a full understanding of the impacts of biological and environmental drivers on microbial community structure and function is often obscured by inherent complexities, including the presence of higher trophic levels and daily/seasonal variations in major environmental drivers.

Lakes and reservoirs have broad distribution, forming a network aquatic habitats across diverse landscapes that can be exploited as sensors of climate change. Meromictic lakes are common throughout the world, and particularly prevalent in polar regions (Cavicchioli, 2015). Meromictic lakes in the Arctic and Antarctic are typically pristine relative to most lower latitude lakes and therefore represent highly sensitive end members of this network (Morgan-Kiss and Dolhi, 2012). Unlike the majority of lakes on earth, the water columns of many polar lakes exhibit minimal seasonal mixing and year-round high physical stability due in large part to the presence of perennial or seasonal ice-covers and prominent haloclines. Microbial communities are spatially stratified, supporting a rich diversity of metabolic processes and adaptation to vastly different habitats within the same water column (e.g., oxic mixolimnion overlaying anoxic monimolimnion; freshwater surface waters vs. hypersaline deep waters) (Spigel and Priscu, 1998). Thus, Antarctic meromictic lakes are important sentinels of climate change and represent unique opportunities for investigating the impact of environmental drivers on microbial community structure and interactions within the microbial loop (Van Der Gucht et al., 2007; Eiler et al., 2012; Gulati et al., 2017).

Antarctica harbors numerous marine-derived, permanently ice-covered meromictic lakes in the ice-free polar deserts, such as Vestfold Hills in Princess Elizabeth Land and the McMurdo Dry Valleys (MDV) in Southern Victoria Land. Lakes in the Vestfold Hills have received intensive investigation to describe the structure and functional potential of microbial populations (Lauro et al., 2011; Laybourn-Parry and Bell, 2014; Cavicchioli, 2015). Microbial community structure within the water columns of the MDV lakes is significantly less understood; although, long term experiments have been conducted on biogeochemical processes and microbial production since the establishment of the McMurdo Long Term Ecological Research (MCM-LTER) site in 1993. Thus, the MDV lakes are poised for studies focused on linking biogeochemistry with microbial community structure and function.

The MDV is a polar desert with average air temperatures of -20°C and precipitation rates of <10 cm per year (Reynolds et al., 1983; Priscu et al., 1998; Chela-Flores, 2011). Photosynthetically available radiation (PAR), is strongly attenuated by the presence of the permanent ice covers causing the shallow layers to have moderate to low solar irradiance and extremely low nutrient levels. The amount of available light rapidly declines with depth, while nutrient concentration and salinity increase steeply at and below the permanent chemoclines. Maximum salinity varies widely across the MDV lakes: while Lake Fryxell is brackish, Lake Bonney exhibits hypersalinity (PSU > 150) at the bottom of the water column (Spigel and Priscu, 1998; Lyons et al., 2000; Doran et al., 2010). Yearly production among the MDV lakes is low; however, there is high variability across the water columns owing to lake-specific variations in nutrients.

Despite the relative stability of environmental influences on the lake microbial communities, the MDVs are experiencing climate related change which is expected have profound impacts on the biota. Following a period of decadal cooling, since a record warm and sunny summer in 2002 the MDV has experienced thinning ice covers, higher stream flow, and significant lake level rise (Doran et al., 2002; Foreman et al., 2004). These climate drivers impart local habitat influences, such as stimulation in production and shifts in diversity of shallow phytoplankton communities of Lake Bonney in response to increased nutrient availability (Priscu, 1995; Teufel et al., 2017). As the extent of ice cover directly influences the availability of underwater PAR, phytoplankton productivity is also inversely related to ice thickness, connecting higher air temperatures in the summer with increased primary production (Obryk et al., 2016). These local climate-related changes in microbial community production and biodiversity are superimposed by ecosystem level changes, including increased connectivity across the discrete landscape units, such as enhanced interactions between soils, streams and lakes (Gooseff et al., 2011). It is anticipated that enhanced connectivity and exchange of biota and materials will result in losses in ecological heterogeneity across the landscape units and reduced community resilience to environmental perturbation (Sokol et al., 2017). Thus, there is an urgency to improve our understanding of the microbial community function within the MDV lakes.

The MDV lake food web is dominated by microorganisms and shows a general absence of metazoans with the exception of low densities of small invertebrates (i.e., copepods, rotifers, tardigrades). Biological communities residing in the MDV lakes can be categorized into four main zones: (i) a planktonic community dominated by photosynthetic protists in the oxygenated photic zone of mixolimnion; (ii) a zone of elevated microbial activity and deep chlorophyll maximum (DCM) within the chemoclines; (iii) a microbial community residing in suboxic/anoxic monimolimnia; and (iv) benthic cyanobacterial mat communities within the littoral zone above the chemoclines. While a number of molecular studies provided early insights on the microbial community structures in MDV lakes, past work was either limited to clone library sequencing (Gordon et al., 2000; Bielewicz et al., 2011; Kong et al., 2012, 2014; Dolhi et al., 2015) or next generation sequencing (NGS) of a limited number of depths of the water columns (Vick-Majors et al., 2014). One recent paper used NGS of 16S rRNA across five MDV lakes (Kwon et al., 2017) but did not consider the microbial eukaryote community. Microbial eukaryotes occupy critical roles in the MDV food web as both the major primary producers and the top predators in the trophic cascade (Priscu et al., 1999; Bielewicz et al., 2011).

This present study extends the current view of the MDV lake microbial communities by examining both bacterial and eukaryal community structure (using Illumina NGS 16S and 18S rRNA gene amplicon sequencing) across a larger number of replicated samples collected from the entire water column of three thoroughly characterized MDV lakes (east and west Lake Bonney, ELB, WLB; Lake Fryxell, FRX). Our approached allowed us to investigate potential correlation patterns between microbes and examine the impact of biogeochemical gradients on the structure of the microbial eukaryote and bacterial communities. Based on earlier evidence of environmental drivers on several functional groups (Laybourn-Parry et al., 1995, 2005; Kong et al., 2012; Li et al., 2019), we hypothesized: (1) owing to relatively low connectivity, microbial community structure, and function will be distinct and niche-specific across the lakes; (2) environmental drivers of microbial community structure will vary between shallow and deep zones of the water columns; (3) interactions among the MDV lake microorganisms will form lake-specific functional groups.

## Materials and Methods

### Site Description and Sample Collection

Water column samples were collected during the field season from November to December 2014 according to Dolhi et al. (2015). Briefly, we collected water samples for DNA extraction through boreholes in the ice cover of the three lakes (Supplementary Figure S1; GPS coordinates: ELB – 77°44′S 162°10′E; WLB – 77°43′S 162°17′E; FRX – 77°37′S 163°11′E) using a 5 L Niskin bottle (General Oceanics, FL, United States). All sampling depths were measured from the piezometric water level in the ice hole using a depth-calibrated hand winch. Sampling depths in the water column were selected to represent major chlorophyll a (*chl-a*) maxima (Dolhi et al., 2015) as well as the epilimnion, thermocline and hypolimnion. Duplicated samples (0.75–2 L of water) from each depth were gently vacuum filtered (0.3 mBar) onto 47 mm Pall Supor^®^ 450 polyethersulfone membranes (Pall Corporation, NY, United States) according to Kong et al. (2012). Filters were immediately flash frozen in liquid N_2_, shipped to a United States laboratory on dry ice and stored at -80°C prior to DNA extraction.

### Environmental Parameter Measurements

Photosynthetically active radiation (PAR), temperature, *chl-a*, primary productivity (PPR), and nutrient (NH_4_^+^, NO_3_^-^, and PO_4_^3-^) concentrations were determined throughout the water columns of ELB, WLB, and FRX. We measured PAR with a Li-Cor LI-193 spherical quantum sensor (Li-COR Biosciences, NE, United States) following Spigel and Priscu (1998), and measured temperature with an SBE 25plus Sealogger CTD profiler (Sea-Bird Electronics Inc., WA, United States). Additionally, *in situ Chl-a* concentration was quantified using a diving spectral fluorometer, FluoroProbe (BBE Moldaenke) according to Dolhi et al. (2015). Light mediated primary production rate (PPR) was determined by measuring NaH^14^CO_3_ incorporation in duplicates over a 24 h *in situ* incubation. Inorganic nitrogen species were determined using a Lachat autoanalyzer and soluble reactive phosphorus (SRP) was manually measured utilizing the antimony-molybdate method (Strickland and Parsons, 1972). PPR and nutrients were measured as part of the NSF-funded McMurdo LTER program according to the methodology outlined in the McMurdo LTER manual^1^.

### Cell Enumeration

Enumeration of free-living bacterial and single cellular eukaryotic cells was carried out using the methods described in Sherr et al. (1993) and Lebaron et al. (1994), respectively. Water samples were first fixed with paraformaldehyde (1% final concentration) and stored at 4°C. Prior to epifluorescent microscopic enumeration, 4′,6-diamidino-2-phenylindole (DAPI, CAS 28718-90-3, Sigma-Aldrich, MO, United States) was added to the samples at the final concentration of 10 ng mL^-1^ for DNA staining, followed by filtering 2 and 50 mL of samples on 25 mm black polycarbonate filters with pore sizes of 0.2 and 0.8 μm (Whatman, United Kingdom) for bacterial and protistan cell counts, respectively. Filters were examined using an Olympus AX-70 Multi-mode System with a specific filter set (EX 360/40 nm, EM 460/50 nm). For each filter, at least 15 random views were recorded using a digital camera (Roper 4k cooled CCD camera, Photometrics, AZ, United States). Images were then calibrated and cell counts were calculated using ImageJ software (V1.47, National Institutes of Health, United States).

### Scanning Electron Microscopy

The procedures for scanning electron microscopy of microbial eukaryotes were previously described in Li et al. (2016). Briefly, water samples from selected depths in ELB (15 and 18 m), WLB (18 and 20 m) and FRX (9 and 11 m) were fixed with 1% paraformaldehyde and 1.25% glutaraldehyde. A secondary fixation of 1% osmium tetroxide was then applied. Specimens were dehydrated through an ethanol series, critical-point dried with CO_2_, and sputter-coated with gold. We examined the morphological characteristics of protists with a Zeiss SUPRA-35 FEG scanning electron microscope (Carl Zeiss Microscopy GmbH, Germany).

### DNA Library Preparation and Illumina MiSeq Sequencing

To isolate environmental DNA from whole filters we used the MP FastDNA^TM^ SPIN DNA kit (MP Biomedicals, CA, United States) following the manufacturer’s instructions and in accordance with Bielewicz et al. (2011). Once isolated, DNA concentration was measured with a NanoDrop 2000 spectrophotometer (Thermo Fisher Scientific, DE, United States) and if needed, DNA was diluted to the appropriate concentration for efficient PCR amplification. Two hypervariable regions, V4 of the 16S rRNA gene (bacteria) and V9 of the 18S rRNA gene (eukaryotes), were amplified using the primer sets which encode F515/R806 for bacteria and F1391/R1501 for eukaryotes, including the barcodes and linkers (Amaral-Zettler et al., 2009; Caporaso et al., 2011). Both PCR and MiSeq sequencing reactions were performed strictly following protocols provided by the Earth Microbiome Project^2^ (Caporaso et al., 2012). Following the manufacturer’s recommendations, we sequenced samples in-house using a 300-cycle MiSeq Reagent Kit v2 (Illumina, CA, United States) on a MiSeq platform with a 2 × 150 bp paired-end run in the presence of 25% PhiX sequencing control DNA.

### Sequence Analysis

To analyze sequences generated on the MiSeq the Quantitative Insights Into Microbial Ecology (MacQIIME v 1.9.1) pipeline (Caporaso et al., 2010) was utilized. Specifically, paired-end reads were randomly subsampled to 5,000 and 10,000 sequences per sample for 16S and 18S rRNA gene sequences, respectively. The protocol retained reads with a minimum quality score of 25 and then used the fast-join method (Aronesty, 2011) for stitching the paired-end reads. Selection of operational taxonomic unit (OTU) and taxonomic classification were both performed following the open-reference clustering procedure. In particular, sequences were clustered against the SILVA (v 123) database using the UCLUST algorithm with a similarity cutoff of 97%. Sequences without any hits from the reference databases were clustered *de novo* to assign new OTUs (Edgar, 2010; Rideout et al., 2014). OTUs with one sequence in samples were discarded to reduce the potential diversity inflation due to sequencing errors.

### Diversity Assessment

For bacteria and eukaryotes, we quantified alpha diversity (number of OTUs, chao1, and Shannon index) in QIIME (Kuczynski et al., 2012) and calculated beta diversity using Bray-Curtis dissimilarity metrics in square-root-transformed OTU counts (Bray and Curtis, 1957; Kuczynski et al., 2012). Nonmetric multidimensional scaling (nMDS) was used to assess the relationship between microbial communities (Ter Braak and Verdonschot, 1995). To test the significance of microbial communities and environmental factors, a one-way analysis of similarity (ANOSIM) with 10,000 permutations (Anderson, 2001; Anderson et al., 2011) was calculated. Further, canonical correspondence analysis (CCA) was employed to investigate the correlation between environmental factor gradients (i.e., light, temperature, salinity, oxygen level, and nutrients, etc.) and the identified communities among the study lakes (Palmer, 1993). Statistics were carried out in the PAST (v 3.07, Oslo, Norway) software (Hammer et al., 2001).

### Network Analysis

For network inference, potential co-occurrence between OTUs was assessed in CoNet (v 1.1.0 beta) (Faust et al., 2012) and the results were visualized in Cytoscape (v 3.4.0) (Shannon et al., 2003). Prior to importing the dataset into CoNet, OTU counts of duplicated samples were averaged and square-root transformed. We then generated co-occurrence networks on (1) 16S and 18S OTUs from all three lakes pooled together; (2) 16S OTUs in Lake Bonney oxic zone and anoxic zones; (3) 16S OTUs in Lake Fryxell. Pairwise scores between OTUs were produced using based on Pearson correlations with *r* = 0.8 and *p* < 0.05. To alleviate compositional bias, 1000 renormalized permutations and bootstraps were generated using the Benjamini-Hochberg *p* value <0.05 threshold, following the ReBoot procedure (Faust et al., 2012).

## Results

### Lake Biogeochemistry

**FIGURE 1:**
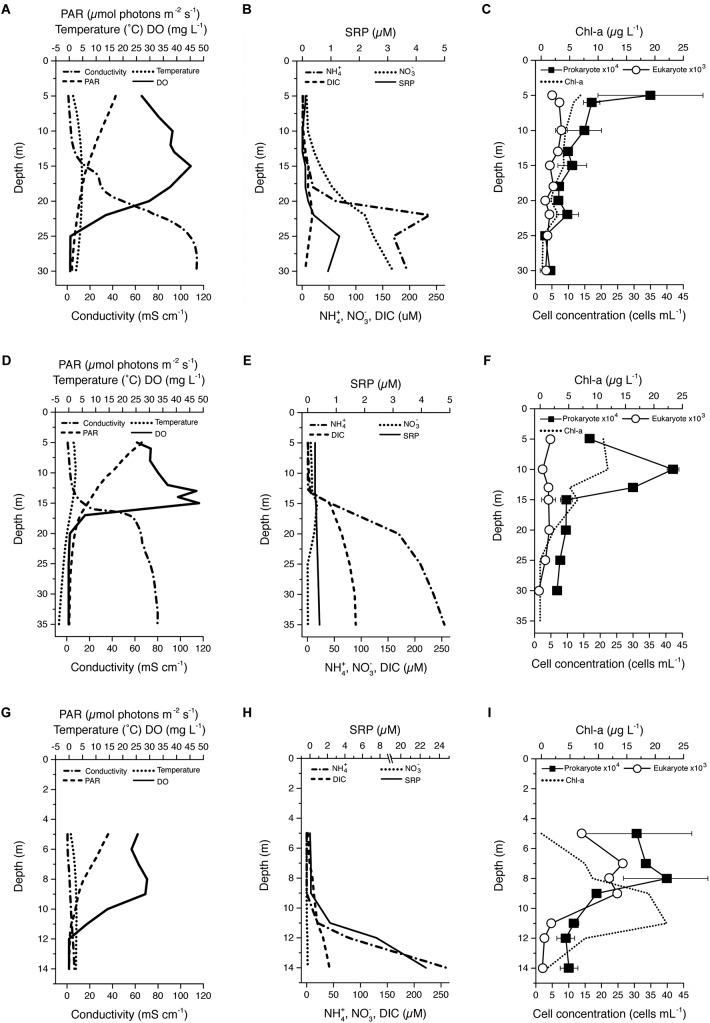
Vertical profiles of selected physicochemical and biological parameters within the lake water columns: East **(A–C)** and West **(C–E)** Lake Boney and Lake Fryxell **(G–I)**. PAR, photosynthetically active radiation; DO, dissolved oxygen; SRP, soluble reactive phosphorus; DIC, dissolved inorganic carbon; Chl-a, chlorophyll a. Data points and bars in **C**, **F** and **I** represent mean ± range (*n* = 2) of cell counts. Bars are not visible when variation between replicates are smaller than the size of the data symbols.

Our study sites included Lake Fryxell and the east and west lobes of Lake Bonney in Victoria Land, Antarctica (Supplementary Figure S1). Lake Fryxell is located at the lower end of the Taylor Valley, between Canada and Commonwealth Glaciers. FRX is a shallow body of water (maximum depth 20 m) and is fed by 13 streams for 4–12 weeks during the austral summer. Lake Bonney is located at the western end of Taylor Valley and is divided into two basins (west and east) by a sill. The shallow waters (above ∼13 m) of the two basins interacted through a narrow channel which is liquid during the summer. ELB receives surface flow from WLB for a short period time during the austral summer. There is also evidence that deeper saline water from WLB has mixed into ELB in the past (Spigel and Priscu, 1998; Doran et al., 2014). The Taylor Glacier as well as a novel geochemical feature, Blood Falls, is connected to WLB. Compared with FRX, significantly fewer streams flow into the Lake Bonney basins (two to WLB and seven to ELB); however, the Lake Bonney streams transport more sediment and salts than those of the FRX basin (Welch et al., 2010). The water columns of ELB, WLB, and FRX exhibited high spatial stratification for major physical and chemical parameters (Figure 1). Permanent chemoclines in these lakes were located at depths of 18–25, 15–18, and 10–12 m for ELB, WLB, and FRX, respectively (Figure 1A,D,G). Above the chemocline, the water columns were generally oligotrophic, in particular phosphorus is extremely limiting in both lobes of Lake Bonney (Priscu, 1995; Dolhi et al., 2015; Teufel et al., 2017). Nutrient ratios within these lakes were generally unbalanced relative to the Redfield ratio (Redfield, 1934; Martiny et al., 2014) (Figure 1B,E,H). PAR in all lakes within the water columns was <10% of ambient incident, with FRX exhibiting the lowest PAR. Conductivity was significantly higher in ELB and WLB compared with FRX (Figure 1A,D,G), while DIC was low in ELB relative to WLB and FRX (Figure 1B,E,H). Last, ELB exhibited a large oxic zone which extended the full sampling area in this study (30 m). In contrast, both WLB and FRX had substantial anoxic zones, starting at 20 and 12 m in WLB and FRX, respectively (Figure 1A,D,G).

### Chlorophyll and Microbial Numbers

Both ELB and WLB exhibited *chl-a* peaks at 5–6 m under the ice cover and a deep *chl-a* peak at a depth of 15 m (ELB and WLB) and 23 m (ELB only) (Figure 1C,F). FRX exhibited a broad *chl-a* peak at 9–11 m and had the concentrations more than twice as high as ELB or WLB (23 μg *Chl-a* L^-1^) (Figure 1I).

Vertical trends in bacterial and eukaryal cell counts were lake specific (Figure 1C,F,I). Eukaryote cell densities in ELB and WLB were low (maximum eukaryote density was <2 × 10^3^ cells ml^-1^) relative to FRX (maximum eukaryote density was >1 × 10^4^ cells ml^-1^). In contrast, bacterial cell density was a comparable range (up to 2 × 10^5^ cells ml^-1^) across all three MDV lakes. Vertical trends in bacterial and eukaryal numbers were similar in FRX, peaking between 5 and 8 m (Figure 1I). However, peaks in bacterial cell numbers did not match that of the eukaryotes and occurred at 5 and 10 m in ELB and WLB (Figure 1C,F).

### PCR-Amplicon Sequencing Summary

After demultiplexing and initial quality filtering, we obtained 6,411,268 and 1,962,313 reads for 16S and 18S rRNA genes, respectively. The sequences were clustered into OTUs using an open OTU selection process, which generated 1138 bacterial and 320 eukaryal OTUs. Known chloroplast sequences were filtered from the 16S rRNA OTUs to reduce their influence in estimating the richness of the samples. Rarefaction analysis based on OTUs indicated that most libraries reached the plateau level (data not shown). The number of bacterial OTUs per sample ranged from 173 to 428 for observed and 218 to 623 for Chao1 estimates, whereas the number of eukaryotic species per sample for observed ranged from 38 to 125 and from 71 to 255 for estimated. At the level of community richness among the three lakes, FRX exhibited higher bacterial species richness than either ELB or WLB in observed species numbers (Tukey’s HSD test, *p* < 0.05) while FRX only showed higher richness than ELB in estimated species numbers (Tukey’s HSD test, *p* < 0.05). No other significant differences were found within pairwise comparison between the lakes (Supplementary Table S1). Relative abundance of unclassified bacterial OTUs ranged from 0.9 to 12%, while unclassified eukaryotic sequences showed relative abundances between 1 and 15% of total OTUs across individual samples. Deeper samples at ELB (30 m) showed higher percentages of unclassified bacterial OTUs whereas shallower samples (ELB 20 m) had higher levels of unclassified eukaryotes. FRX at 14 m showed the highest percentages of both unclassified bacterial and eukaryal OTUs.

### Eukaryal Community Composition

**FIGURE 2:**
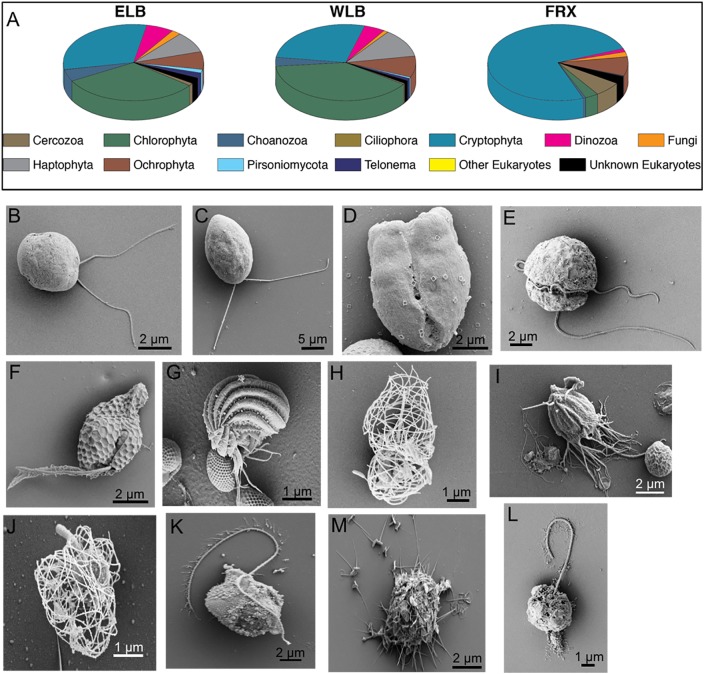
Phylogenetic and morphological diversity of microbial eukaryotes communities residing in the water columns of the east and west lobes of Lake Bonney (ELB and WLB, respectively) and Lake Fryxell (FRX). **(A)** Phylogenetic diversity based on 18S rRNA gene sequences integrated within the whole water column of individual lakes. **(B–M)** Representative SEMs of key protists residing in the MDV lakes. **B**, Haptophyta, *Isochrysis* sp. (ELB,WLB); **C**, Chlorophyta, *Chlamydomonas* sp. (ELB, WLB); **D**, Chlorophyta, prasinophyte (ELB); **E**, Dinozoa (ELB); **F**, Cryptophyta, *Geminigera* sp. (FRX); **G**, Ciliophora, *Euplotes* sp. (FRX); **H–J**, Choanozoa (ELB, WLB); **K,M**, Ochrophyta, Chrysophytes (ELB, WLB); **L**, Personiomycota, *Personia*, sp. (ELB, WLB).

While there have been a few reports on bacterial community structure in the MDV lakes (Vick-Majors et al., 2014; Kwon et al., 2017), a thorough understanding of the MDV lake eukaryal communities has been lacking. We combined NGS sequencing of the V9 hypervariable region of the 18S rRNA gene (Figure 2A, 3A–C) with a morphological survey of key eukaryotes in Lake Bonney and Fryxell using SEM (Figure 2B–M). This combined approach revealed a diverse community of eukaryotes with various potential functional roles (photosynthetic, mixotrophic, phagotrophic) in the MDV food web.

**FIGURE 3:**
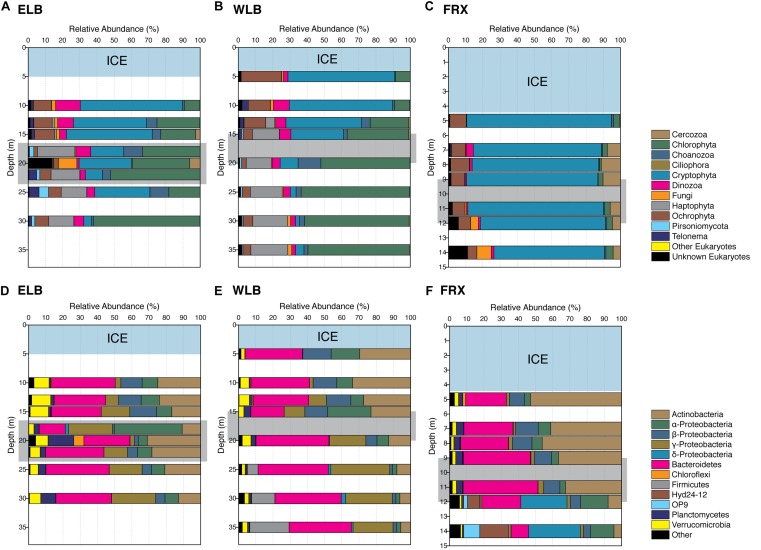
Diversity of Eukarya **(A–C)** and Bacteria **(D–F)** in the east and west lobes of Lake Bonney (ELB and WLB, respectively) and Lake Fryxell (FRX). The counts of OTUs based on 16S and 18S rRNA gene sequences are binned to phylum level, except for Proteobacteria which are shown in families. Light blue shows perennial ice covers and light gray indicates the permanent chemoclines in each lake.

A total of 12 phyla were detected among the microbial eukaryote communities (Figure 2A). Dominant eukaryote phyla across all the lakes were Chlorophyta, Cryptophyta, Haptophyta, and Ochrophyta. In both lobes of Lake Bonney, Cryptophyta and Chlorophyta were the most abundant, while cryptophytes made up over 74% of the total eukaryote community in FRX (Figure 2A). The most abundant OTUs among the Chlorophyta genera in ELB and WLB were related to nonmotile picoeukaryote, *Mychonastes*, and a biflagellate, *Chlamydomonas* (Figure 2C). This agreed with previous studies that the shallow zone of ELB is dominated by *Chlamydomonas* sp. ICE-MDV (Bielewicz et al., 2011; Kong et al., 2012; Dolhi et al., 2015). A third chlorophyte, a flagellated prasinophyte related to *Pyramimonas*, was detected in ELB only (Figure 2D). A nanoflagellate related to the genus *Isochrysis* dominated the haptophyte group of ELB and WLB (Figure 2A,B). Dinoflagellates (Figure 2E) were present in higher abundance in ELB and WLB, relative to FRX (Figure 3A). Previous studies reported that a mixotrophic *Isochrysis* community plays a significant role in primary production in Lake Bonney, but is absent from Lake Fryxell (Figure 2A,B) (Kong et al., 2012; Li et al., 2016). In contrast, sequences related a second mixotroph, *Geminigera*, dominated the cryptophyte group across all three lakes (Figure 2A,F). Earlier studies observed that FRX is dominated by mixotrophic cryptophytes which predate heavily on bacterioplankton (Roberts and Laybourn-Parry, 1999). A diverse group of eukaryotes related to the stramenopile supergroup were also detected in ELB and WLB (Figure 2A), including a parasitic nanoflagellate related to *Personia* sp. (Figure 2L; Li et al., 2016) and a heterotrophic chrysophyte related to *Paraphysomonas* (Figure 2M). Other heterotrophic protists including a ciliate related to *Euplotes* (Figure 2G). Several choanoflagellates related to *Stephanoeca* (Figure 2H,J) and *Monosigma* (Figure 2I) were also observed in all three lakes, albeit at low levels (<5% total eukaryote population; Figure 2A).

Microbial eukaryote community composition varied with depth in ELB and WLB (Figure 3A,B). Chlorophytes were the most abundant organisms within the both lobes contributing 30.5 and 38.5% of total sequences for ELB and WLB, respectively (Figure 2A). Haptophyte sequences were detected in all layers of ELB and WLB, but were most abundant in depths at and below the chemocline (Figure 3A,B). Cryptophyta was abundant (52–70% of the eukaryal community) in shallow layers of ELB and WLB (5–13 m), but declined at the chemocline (15 m) and were rare in deeper layers of the water columns of both lobes (Figure 3A,B). This agreed with previous reports on vertical trends in cryptophyte *rbcL* expression and abundance (Kong et al., 2012; Dolhi et al., 2015). Ochrophyta sequences related to a non-pigmented Chrysophyte (*Paraphysomonas*) were detected throughout the water columns of ELB and WLB, with peaks in abundance near the bottom of the chemocline in both lobes (Figure 3A,B). Choanoflagellates were also present in low abundance throughout the water columns and peaked in the chemoclines of both lobes (Figure 3A,B).

In contrast with Lake Bonney communities, the FRX eukaryotic community was mostly comprised of Cryptophyta throughout the water column, representing 64–84% of the total eukaryote sequences (Figure 2A, 3C). This agreed with a recent report on the vertical distribution of cryptophyte *rbcL* gene copy numbers in FRX which were ∼100-fold higher compared with ELB and WLB (Dolhi et al., 2015). Ochrophyta were the second most abundant eukaryote group in FRX samples, and included numbers of sequences related to Chrysophytes (i.e., *Cyclonexis* and *Paraphysomonas*) and a nonmotile picophytoplankton, *Nannochloropsis*, representing 7.9 and 1% of total eukaryotic sequences, respectively. In agreement with a previous reports (Vincent, 1981; Dolhi et al., 2015), two flagellates, *Pyraminonas* and *Chlamydomonas*, dominated chlorophyte group in FRX.

### Bacterial Community Diversity

We assessed bacterial community diversity by sequencing the V4 region of the 16S rRNA gene. On average only 0.09% of the total OTUs identified were Archaea, possibly due to the nature abundance or bacterial-domain-specific primer sets used in this study (Baker et al., 2003). Therefore, in order to avoid potential bias, we excluded all Archaean OTUs from our community structure analysis. Phylotyping of the bacterial communities from the water columns of ELB, WLB, and FRX revealed that the most abundant OTUs in the MDV lake communities belonged to the phyla *Bacteroidetes*, *Actinobacteria* and *Proteobacteria*, which together contributed over 85% of the total OTU counts in each lake (Figure 3D–F). We found that 13 phyla were common to all three lakes, with three phyla (*Tenericutes*, *Spirochaetes* and *OP9* clade) only found in WLB and FRX, and two distinct phyla (*SR1* and *GN02* clades) shared between ELB and FRX. FRX exhibited the highest number of unique bacterial phyla, where of the 28 phyla identified, nine (*Armatimonadetes*, *Caldiserica*, *Lentisphaerae*, *GN04*, *Hyd24-12*, *NKB19*, *OP3*, *WS1*, and *WWE1*) were only present in FRX samples (Figure 3F).

Distribution of major bacterial phyla within the ELB water column was roughly comparable across the sampling depths, with the exception of 18 m where abundance of OTUs (24.6% of total OTUs) belonging to *Rhodobacteraceae* family in *Alphaproteobacteria* exhibited significantly higher than that (<1%) in other layers of the water column (Figure 3D). In contrast with ELB, bacterial communities from WLB and FRX exhibited depth-specific changes in the distribution of major phyla (Figure 3E,F). The dominant phyla in WLB were segregated by the chemocline; specifically, *Actinobacteria* were abundant in the upper layers and were replaced by *Gammaproteobacteria* below the chemocline (Figure 3E). A marked increase in abundance of *Fimicutes* occurred in the oxic-anoxic transition zone (25 m) and anoxic zone (30 and 35 m) of WLB, which agreed with a recent report on bacterial distribution across several MDV lakes (Kwon et al., 2017). In the oxic zone of FRX (5–11 m), *Bacteroidetes* (32 ± 8%) and *Actinobacteria* (42 ± 8%) were dominant, while their relative abundance declined to 10 and 6%, respectively, in the anoxic zone (12 and 14 m). In the deep anoxic layers of FRX, *Deltaproteobacteria*, *Hyd24-12*, and *OP9* (Atribacteria) increased in relative abundance (Figure 3F). Thus, the distribution of major bacterial phyla in ELB was more uniform throughout the highly oxic water column relative to the other lakes, while the anoxic zones of WLB and FRX harbored unique communities.

### Microbial Community Structure

**FIGURE 4:**
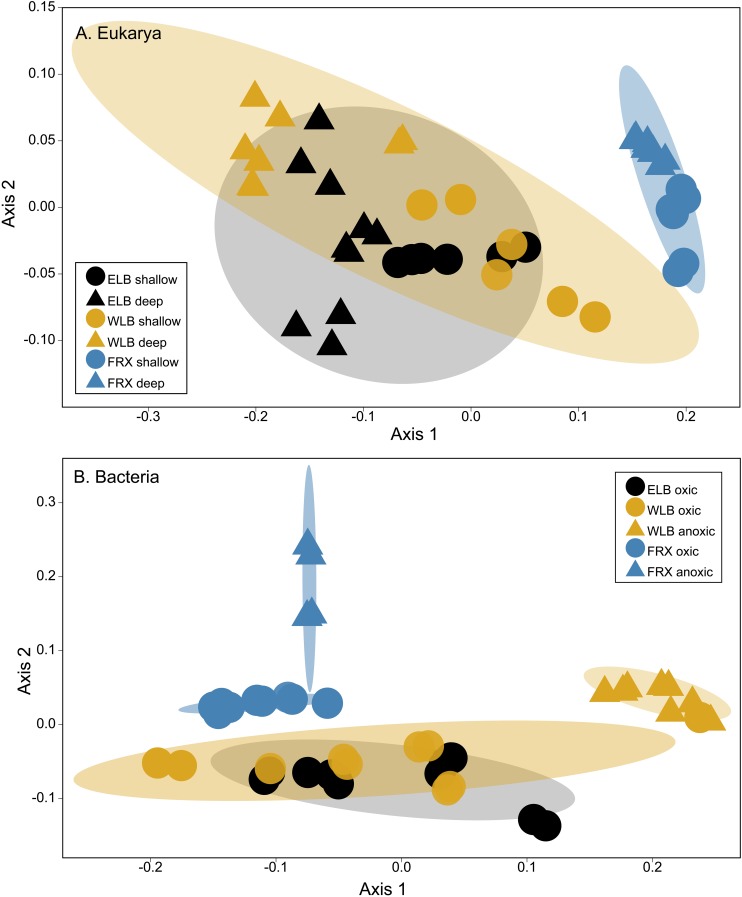
**(A)** A non-metric multidimensional scaling (NMDS) plot of eukaryotic communities (OTU counts, Bray-Curtis dissimilarity, stress = 0.077). Shaded areas indicate 90% similarity within each lake. Shallow: epilimnion. Deep: chemocline + hypolimnion. **(B)** A NMDS plot of bacterial communities (OTU counts, Bray-Curtis dissimilarity, stress = 0.072). Oxic and anoxic: upper oxic zone and deep anoxic zone in the water columns, respectively. Shaded areas indicate 75% similarity within each lake.

Patterns of eukaryal and bacterial community structure were examined using non-metric multidimensional scaling based on Bray-Curtis dissimilarity metrics (Figure 4). The ordination based on the Bray-Curtis dissimilarity matrix indicated that both ELB and WLB eukaryote communities clustered closely (Figure 4A). Eukaryote diversity and abundance in FRX was largely distinct from ELB or WLB (ANOSIM *p* < 0.001). When comparing ELB and WLB communities, despite ANOSIM *p* < 0.05, the small R value (0.293) indicated microorganism combination dissimilarity between the lakes was just slightly greater than within the lakes. Our results also indicated that eukaryotic communities in the freshwater mixolimnion were significantly different to communities associated with either the chemocline or monimolimnion (Supplementary Table S2).

Clustering of bacterial communities was lake-specific and separated by oxic and anoxic zones (Figure 4B). Bacteria from the upper oxic zones of ELB and WLB clustered together (ANOSIM *R* = 0.06, *p* = 1). Communities from the oxic layers of FRX as well as anoxic areas of FRX and WLB differed significantly (*p* = 0.017) from each other and clustered separately (Supplementary Table S2).

### Correspondences Between Microbial Communities and Environmental Factors

**FIGURE 5:**
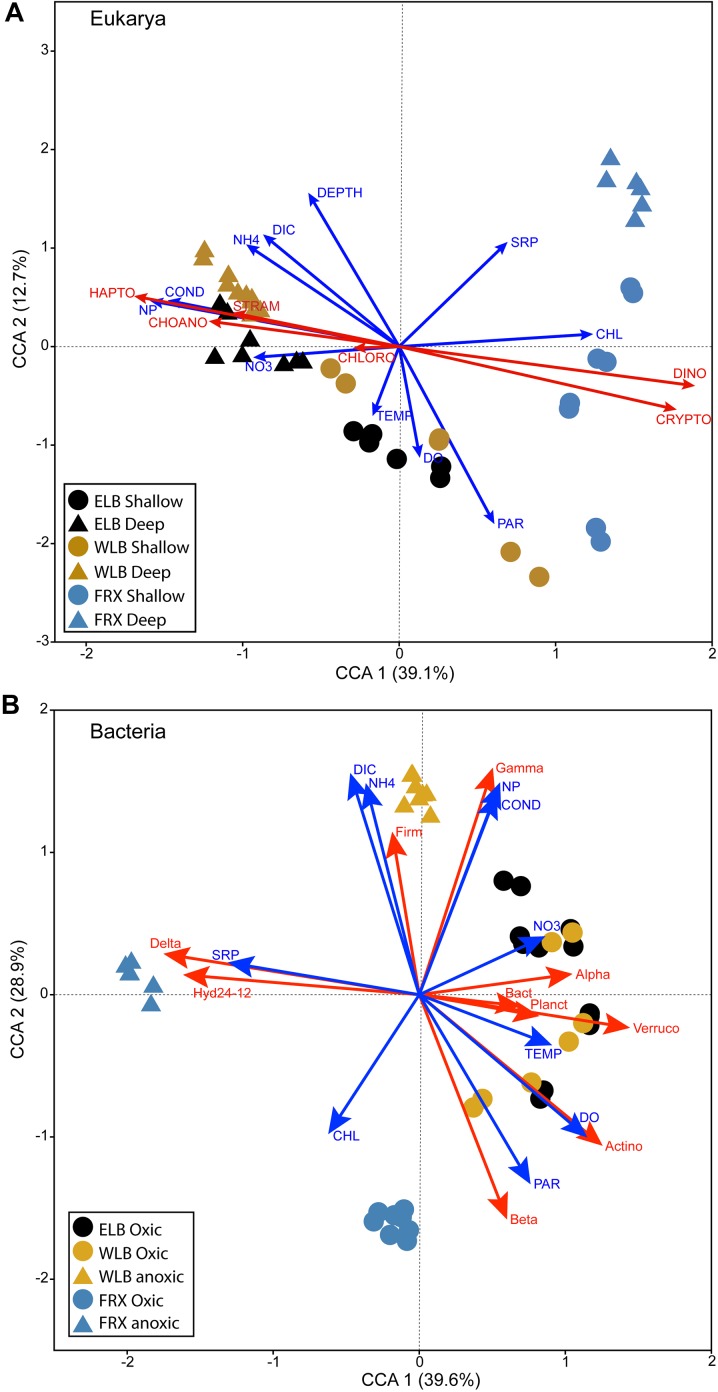
Canonical correspondence analysis (CCA) plots showing corresponding relations between eukaryal **(A)** and bacterial **(B)** communities to physicochemical and biological factors in the east and west lobes of Lake Bonney (ELB and WLB, respectively) and Lake Fryxell (FRX). Shallow: epilimnion; Deep: chemocline + hypolimnion. Oxic and anoxic: upper oxic zone and deep anoxic zone in the water columns, respectively. TEMP, temperature; COND, conductivity; PAR, photosynthetically active radiation; NH_4_, ammonium; NO_3_, nitrate and nitrite; NP, N:P ratio; DO, dissolved oxygen; SRP, soluble reactive phosphorus; DIC, dissolved inorganic carbon; Chl, chlorophyll a. Algal abundance: CHLORO, chlorophytes; CRYPTO, cryptophytes; CHOANO, choanoflagellates; DINO, dinoflagellates; HAPTO, haptophytes. Bacterial abundance: Alpha, Alphaproteobacteria; Beta, Betaproteobacteria; Gamma, Gammaproteobacteria; Delta, Deltaproteobacteria; Actino, Actinobacteria; Bact, Bacteroidetes; Firm, Firmicutes; Planct, Planctomycetes; Verruco, Verrucomicrobia.

We performed CCA to investigate the linkages between microbial communities and the environmental factors in all three studied lakes (Figure 5). In ELB and WLB, distribution of eukaryote communities residing in the shallow water column of ELB and WLB positively corresponded with temperature, PAR and dissolved oxygen; while communities within the deep communities (i.e., at and below the chemoclines) were correlated with conductivity, DIC, and N:P ratio (Figure 5A). FRX eukaryote communities were separated from Lake Bonney by phytoplankton biomass (*chl-a*) and SRP. Last, ELB and WLB were separated from FRX eukaryote communities by the abundance of haptophytes, choanoflagellates, stramenopiles (Ochrophyta), dinoflagellates, and cryptophytes (Figure 5A).

Bacterial communities residing within the oxic layers of Lake Bonney corresponded positively with a number of parameters, including temperature, PAR, dissolved oxygen and nitrate (Figure 5B). In contrast, WLB deep anoxic layers corresponded strongly with conductivity, DIC, NH_4_^+^ and N:P ratio, as well as abundance of Firmicutes and Gammaproteobacteria (Figure 5B). Bacterial communities residing in the oxic layers of FRX were separated from those of Lake Bonney by *chl-a*. SRP concentration corresponded with deeper anoxic layers of FRX where Deltaproteobacteria family and Hyd24-12 clade were abundant.

### Co-occurrence Microbial Patterns

**FIGURE 6:**
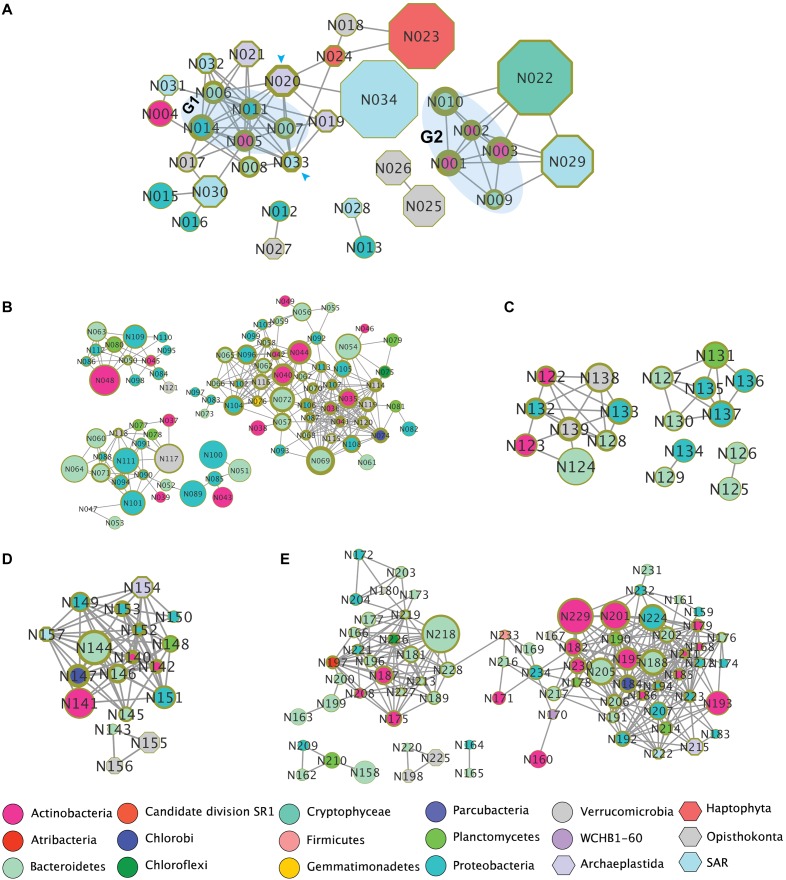
Co-occurrence patterns of OTUs in Lakes Bonney and Fryxell based on pairwise Pearson correlation coefficient between samples. **(A)** Co-occurrence between Eukarya and Bacteria in Lake Bonney. **(B)** Bacteria in Lake Bonney oxic zone. **(C)** Bacteria in Lake Bonney Anoxic zone. **(D)** Co-occurrence between Eukarya and Bacteria in Lake Fryxell. **(E)** All bacterial OTUs in Lake Fryxell. Nodes represent individual OTUs. Node colors represent phyla. The size of each node represents relative abundance of the corresponding OTU. Thickness of the circle outside each note indicates number of edges of corresponding note. Taxonomies of nodes were summarized in Supplementary Table S3.

To explore potential co-occurrence patterns between organisms in the lakes and specific regions (i.e., oxic and anoxic zones), we identified OTUs that presented highly correlative based on Pearson correlation coefficient (PCC *r* > 0.8) between samples and co-occurrence patterns (Figure 6). In both lobes of Lake Bonney, we observed that 16 eukaryotic OTUs co-occurred with 18 bacterial OTUs (Figure 6A). Bacterial OTUs related to Actinobacteria and Bacteroidetes exhibited the highest number interactions with eukaryotes. A chlorophyte OTU-HQ191357.1.3038 (closely related to a *Mychonastes* sp. accession no. AF357153.1) and an Ochrophyta OTU-AF045049.1.1790 (related to *Nannochloropsis salina* accession AF045049) (indicated by arrows in Figure 6A) had the most complicated interactions with nine nodes (OTUs). *Mychonastes* was an abundant Chlorophyte in both lobes of Lake Bonney (Figure 3A,B), while a previous paper reported high abundance of *rbcL* DNA and mRNA related to *Nannochloropsis* in the chemoclines of both lobes of Lake Bonney (Kong et al., 2012). The *Mychonastes* OTU-HQ191357.1.3038 interacted with four eukaryotes (another second *Mychonastes*, two Stramenopiles, *Nannochloropsis*, and a haptophyte), as well as five bacteria. The *Nannochloropsis* OTU-AF045049.1.1790 was correlated with three eukaryotes and six bacteria. Both *Mychonastes* and *Nannochloropsis* OTUs were correlated with a group of five bacterial OTUs (G1 group, indicated by shaded area in Figure 6A) which were closely related to three aerobic chemoheterotrophs including *Balneola alkaliphila* (NR_044367.1, 99% similarity) (Urios et al., 2008), *Rathayibacter festucae* (NR_042574.1, 99%) (Stackebrandt et al., 2007) and *Brumimicrobium mesophilum* (NR_115845.1, 95%) (Yang et al., 2013), an aerobic anoxygenic phototroph *Roseibacterium beibuensis* sp. (NR_132716.1, 97%) (Mao et al., 2012), as well as a likely endosymbiont *Olavius algarvensis* (AJ620496.1, 96%) (Ruehland et al., 2008). In addition, the *Nannochloropsis* OTU-AF045049.1.1790 also co-occurred with an OTU in the family Flavobacteriaceae. The G1 bacteria group also interacted with two *Paraphysomonas* (family Chrysophyceae) and a *Chlorella* OTU (family Trebouxiophyceae). It was also noted that two haptophytes (in families Imantonia and Isochrysidaceae) were correlated with only one bacterial OTU (family Verrucomicrobia) which was closely related to *Luteolibacter algae* (AB677319.1, 97%) that was reported to have the capability of degradation of algal products (Ohshiro et al., 2012). A second bacterial group (G2) was identified as four OTUs belonging to Bacteroidetes and Actinobacteria phyla co-occurred with two additional eukaryotic OTUs (families Cryptomonadales and Dinophyceae). Interestingly, the only bacterial OTU observed to co-occur with a ciliate in Intramacronucleata (*Oligohymenophorea*) was reported as an endosymbiont of an *Euplotes* (Schrallhammer et al., 2013).

As distribution of the bacterial community was strongly influenced by the presence of oxygen, we investigated the bacterial co-occurrence patterns in oxic and anoxic zones of Lake Bonney (Figure 6B,C). The networks contained 87 and 18 nodes for oxic and anoxic zones, respectively. Interconnections in oxic zone (average 7.1 edges per node) was significant higher (*t*-test, *p* < 0.05) than that in anoxic zone (average 3.6 edges per node) (Figure 6B,C). We observed fairly complex subnetworks in the oxic zones including Actinobacteria, Bacteroidetes, Chlorobi, Chloroflexi, Gemmatiminadetes, Planctomycetes, Proteobacteria, and Verrucomicrobia (Figure 6B). Most of the known OTUs in these networks were heterotrophic bacteria, some affiliated with hydrogen-oxidizing *Hydrogenophaga*, an anaerobic syntroph related to *Smithella*, and the endosymbiont *Rickettsia*.

Co-occurrence patterns between eukaryotes and bacteria in FRX were distinct from and generally fewer compared with Lake Bonney (Figure 6A,D). We identified four eukaryotic OTUs affiliated with two choanoflagellates, a chlorophyte (*Chlamydomonas*) and the heliozoan *Hedriocystis* which were positively correlated with 14 bacterial OTUs. Most of the bacteria were related to heterotrophic species. The chlorophyte and Stramenopile OTUs co-occurred with a syntrophic bacterium in genus *Smithella* (OTU-HM128460.1.1462). Two choanoflagellates co-occurred with a *Flavobacterium* (OTU_DQ264580.1.1501) (Figure 6E). In comparison with Lake Bonney, Lake Fryxell bacteria co-occurrence patterns presented more diverse (10 bacterial phyla) and complex interactions (average 8.8 edges per note) (Figure 6E). We observed two major subnetworks and the interactions between them were connected by two OTUs affiliated with Firmicutes and Bacteroidetes.

## Discussion

To date, a full understanding of vertical distribution patterns in microbial communities within the stratified aquatic environment of the MDV lakes has been limited by a lack of sampling depths spanning the full water column, as well as a general absence of 18S rRNA sequences. In this study, we provided new insights into MDV lake microbial community structure and potential environmental and biological drivers of these aquatic ecosystems by utilizing small subunit rRNA gene amplicon sequencing for both bacterial and protistan communities throughout the water columns of three highly studied lakes. Owing to the strong vertical chemical gradients, redox transitions and solar irradiance availability, stratified microbial communities are a major feature of meromictic lakes (Laybourn-Parry et al., 1995; Comeau et al., 2012; Andrei et al., 2015). Our study revealed that MDV lake bacterial and eukaryal communities exhibit vertical stratification and complex distribution patterns which are influenced by multiple physicochemical drivers. Bacterial and eukaryal communities residing in the upper portions of the water columns above the chemoclines of ELB and WLB, which are connected for several weeks in the austral summer, were more similar to each other than within an individual lake basin. In addition, eukaryal communities were less influenced by their location in the water column, while bacteria community structure differed significantly between the upper oxic zones vs. the deep anoxic layers of FRX and WLB.

The structures of the bacterial and eukaryal communities were influenced by different environmental factors. Bacterial communities were distinct at the level of environmental drivers and potential biotic interactions between the oxic and anoxic zones. In contrast, eukaryotic communities exhibited stronger separation on the basis of lake depth; in particular when comparing the shallow mixolimnion to the deeper chemocline and monimolimnion (Figure 3, 4 and Supplementary Table S3). This differential clustering of bacterial vs. eukaryal communities likely reflects the strong influence of oxygen on the bacterial communities vs. light on the eukaryotes.

In agreement with previous reports, distribution of major bacterial phyla was strongly influenced by oxygen (Vick-Majors et al., 2014; Kwon et al., 2017). The three basins differed in the size of their oxic zones which in turn had a significant impact on the distribution of bacterial communities. The water column in ELB is generally highly oxygenated whereas WLB and FRX are anoxic below 25 and 12 m, respectively. Bacterial communities from the oxic zones of the three basins were more similar to each other, while WLB and FRX exhibited very distinct communities residing in the anoxic zones. Comparison of the major phyla in the lakes indicated that although the bacterial groups of *Bacteroidetes*, *Actinobacteria* and *Betaproteobacteria* were more abundant in the oxic zones of all three lakes, the anoxic bacterial communities were highly variable (Figure 3, 5 and Supplementary Table S3). In the WLB anoxic zone, *Gammaproteobacteria* (majorly *Alteromonadaceae* and *Marinobacter*) and *Firmicutes* (majorly *Acidaminobacteraceae*) exhibited relatively high abundance. In contrast, *Deltaproteobacteria* (majorly *Desulfobulbaceae*, *Geobacteraceae*, and *Syntrophaceae*) was present in large numbers in the deep FRX layers. WLB is characterized by a hypersaline deep water layers and the presence of both *Alteromonadaceae* and *Acidaminobacteraceae* was not surprising as these two families are reportedly abundant in other high salinity aquatic environments (Kharroub et al., 2011; López-Pérez and Rodriguez-Valera, 2014; Andrei et al., 2015). The *Alteromonadaceae* family comprises a diverse group of gammaproteobacteria which are mostly marine in origin and require sodium for growth. Many display diverse potential for degrading a variety of substrates, and are often associated with particulate material and marine snow (Ivanova et al., 2004). One Antarctic strain, *Glaciecola punicea*, was associated with sea-ice diatom assemblages (Bowman et al., 1998). *Acidominobacter* is an obligate anaerobe and several strains exhibit the ability to degrade various amino acids and are often associated with hydrogen-consuming organisms (Meijer et al., 1999). These bacteria residing in the deep zones of WLB would have access to a large pool of ancient organic matter (Knoepfle et al., 2009; Khan et al., 2016).

Unlike WLB, the deep layers of the FRX water column have high biogenic methane and reduced forms of sulfur compounds (e.g., hydrogen sulfide and sulfite) (Karr et al., 2006; Sattley and Madigan, 2006). Earlier studies reported that FRX has a robust community of purple non-sulfur bacteria, sulfur oxidizers, and methanogenic Archaea (Karr et al., 2003, 2005, 2006). In this current study, we found that *Desulfobulbaceae* dominated *Deltaproteobacteria* sequences in samples from these layers. The *Desulfobulbaceae* family has been isolated from other anoxic environments and are involved in oxidation of methane or other simple organic carbon molecules in similar conditions (Teske et al., 2002; Lloyd et al., 2006; Kuever, 2014). Members of this group are typically sulfate reducers, and it is likely that this organism may play a role in the active sulfur cycle in FRX. Interestingly, Sattley and Madigan (2010) observed that the carbon substrate preference of the sulfate reducing bacteria (SRB) population residing in FRX differed throughout the water column. Communities residing below the chemocline were stimulated by lactate, while the deeper SRB community exhibited a preference for acetate. Cold adapted acetogenic bacteria were also detected in the sediments of FRX (Sattley and Madigan, 2007). Thus, the dominant organisms detected by this study in deep anoxic waters may form niche specific communities and likely play important roles in biogeochemical cycling. The dissimilarity of bacterial distribution in anoxic waters between WLB and FRX is likely indicative of additional environmental conditions influencing the bacterial community structure in these dark, anoxic environments, including the presence of Blood Falls, a subglacial iron brine input into WLB (Mikucki et al., 2004).

We also noted spatial differences in the eukaryotic community structure within and between the three study lakes (Figure 4, 5 and Supplementary Table S2). Previous work has shown that MDV lake eukaryote populations are dominated by photosynthetic protists (Bielewicz et al., 2011; Kong et al., 2012, 2014; Dolhi et al., 2015), while a recent study reported complex spatial patterns in protist photosynthetic and heterotrophic enzyme activities across four MDV lakes (Li et al., 2019). The pure photoautotroph *Chlamydomonas* was highly abundant in the shallow zones of WLB and ELB but absent from FRX, and declined in deeper waters limited by PAR. Previous work also reported that gene copy abundance and expression of *Chlamydomonas* sp. *rbcL* genes in Lake Bonney exhibited a strong correlation with PAR availability during the summer (Dolhi et al., 2015) and polar night transition (Kong et al., 2012; Morgan-Kiss et al., 2016). A recent paper reported that two ELB *Chlamydomonas* sp., *C.* spp. UWO241 and ICE-MDV, exhibited differential physiologies based on their position in the water column (Cook et al., 2019). In addition, single cell sequencing of Lake Bonney *Chlamydomonas* sp. revealed that they are both prey for a heterotrophic nanoflagellates, *Personia*, and are also associated with unique bacterial communities in comparison with other MDV lake eukaryotes (Li et al., 2016). Thus, the obligate photoautotrophic *Chlamydomonas* spp. is involved in more diverse roles in the food web than previous assumed and have evolved physiological plasticity to exploit specific niches within the Lake Bonney water column.

This study revealed that a previously unidentified Chlorophyte, *Mychonastes*, is abundant throughout the water column of WLB and ELB. *Mychonastes* are nano- or pico-phytoplankton which have been Antarctic soil biocrusts and permafrost (Gilichinsky et al., 2007; Büdel et al., 2016), and the anoxic monimolimnion of a meromictic lake in France (Lepère et al., 2016). We detected high levels of both *Chlamydomonas* and *Mychonastes* in the anoxic zone of WLB, suggesting that these Chlorophyta may exhibit metabolic flexibility and switch to anaerobic energy metabolism under conditions of anoxia and darkness. A genomic survey of several unicellular algae revealed than many are capable of mixed acid fermentation, including the genera *Chlamydomonas* and *Chlorella* (Atteia et al., 2013). This could represent an additional, previously unconsidered, role for the chlorophytes in MDV food web. Furthermore, it may partially explain how obligate photoautotrophs such as *C.* sp. UWO241 and ICE-MDV survive the polar winter (Morgan-Kiss et al., 2016).

In common with the Chlorophytes, cryptophytes were detected in the upper photic zones of WLB and ELB, but also highly abundant throughout the water column of FRX. Since mixotrophic cryptophytes do not solely rely on solar irradiance to provide energy via photosynthesis, they can live in the aphotic zone of the water column by grazing on bacteria (Marshall and Laybourn-Parry, 2002; Laybourn-Parry et al., 2005). The relatively productive FRX would provide significant bacterial prey as an alternative energy source for cryptophytes, while cryptophytes residing in Lake Bonney may rely on phagotrophy to supplement nutrient acquisition in the oligotrophic shallow layers. In addition, tolerance to low oxygen environments and high sulfate concentrations in FRX have probably allowed these organisms to dominate the protist communities in FRX (Gasol et al., 1993). Correspondence analysis in this study indicates that cryptophyte populations are negatively correlated with salinity, which may restrict their distribution to the shallow waters of WLB and ELB (Figure 5). In addition, biotic interactions such as competition between mixotrophs and pure heterotrophs (e.g., choanoflagellates and other heterotrophic nanoflagellates, etc.) could be another constraint on the vertical distribution of the cryptophytes in Lake Bonney (Roberts and Laybourn-Parry, 1999). Supporting this, Chrysophyta (*Paraphysomonas*) dominated the Stramenopile supergroup in both ELB and WLB, showing peak abundance in the chemocline. Previous literature describes Chrysophyta as often associated with suboxic to anoxic habitats and have been found to play important roles in transferring carbon and nutrients from primary and secondary production to higher trophic levels (Stock et al., 2009; Park and Simpson, 2010; Wylezich and Jürgens, 2011). In addition, a second mixotroph, a haptophyte related to the nanoflagellate *Isochrysis*, dominates the chemocline of both WLB and ELB, but is undetectable in FRX (Kong et al., 2012, 2014; Dolhi et al., 2015). It is likely that this haptophyte plays a comparable role in Lake Bonney as cryptophytes play in FRX, that is, the dual roles of both major primary producers and active predators, depending on the local environmental conditions. However, previous work also hypothesized that *Isochrysis* is a major producer of dimethylsulphonioproprionate (DMSP) (Dolhi et al., 2015), suggesting an additional role of biogenic sulfur production for the Lake Bonney haptophye.

## Author Contributions

WL contributed to data acquisition and analyses as well as drafting the figures and manuscript. RM-K contributed to the conception of the project and critical revisions to the work.

## Conflict of Interest Statement

The authors declare that the research was conducted in the absence of any commercial or financial relationships that could be construed as a potential conflict of interest.

## Footnotes

1 http://mcm.lternet.edu

2 http://www.earthmicrobiome.org
