# Supplementary material for: Influence of Environmental Drivers and Potential Interactions on the Distribution of Microbial Communities From Three Permanently Stratified Antarctic Lakes
Source: Front Microbiol. 2019 May 15;10:1067. doi: 10.3389/fmicb.2019.01067 (PMC6530420; doi:10.3389/fmicb.2019.01067)
Supplement: Supplementary file 1 [file Data_Sheet_1.docx]

Figure S1. Map of Antarctica showing the general location of the McMurdo Dry Valleys. Satellite imagery of Taylor Valley where study sites are located: permanently ice-covered Lake Bonney east (ELB) and west (WLB) lobes and Lake Fryxell (FRX). Image was edited from a LIMA based map from the Antarctic Geospatial Information Center (<http://www.agic.umn.edu/maps/Antarctic>).


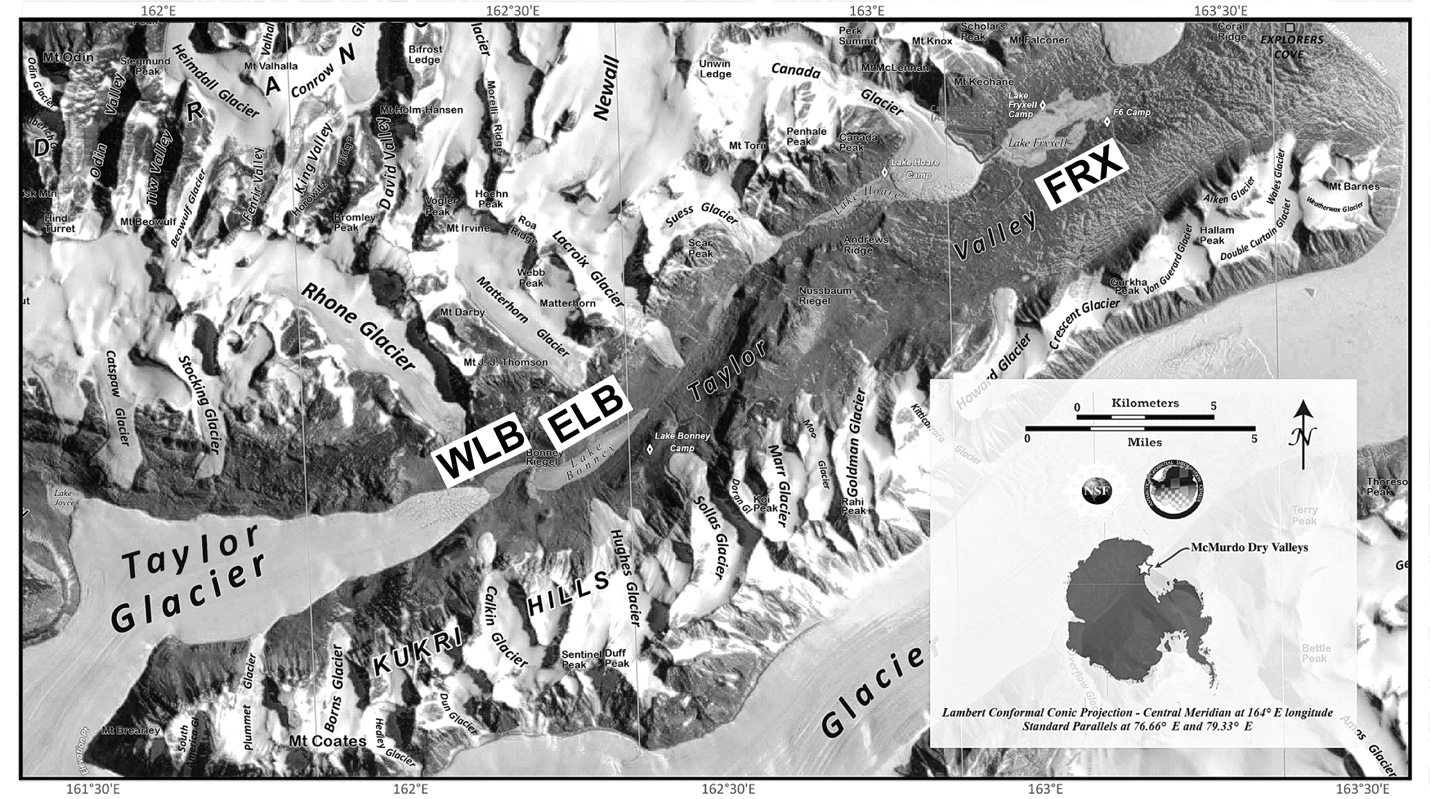


Table S1. Pairwise comparison of species richness between lakes using Tukey’s HSD test.

* p-value less than 0.05

| Pairs | Bacteria (observed) | | Bacteria (estimated) | | Eukaryotes (observed) | | Eukaryotes (estimated) | |
| --- | --- | --- | --- | --- | --- | --- | --- | --- |
|  | Q score | p-value | Q score | p-value | Q score | p-value | Q score | p-value |
| ELB-WLB | 0.956 | 0.779 | 1.733 | 0.445 | 2.257 | 0.259 | 2.370 | 0.226 |
| ELB-FRX | 4.984 | *0.003 | 3.571 | *0.040 | 0.072 | 0.999 | 1.887 | 0.385 |
| WLB-FRX | 4.028 | *0.018 | 1.837 | 0.404 | 2.329 | 0.238 | 0.483 | 0.938 |

Table S2. ANOSIM of communities from the studied lakes and sampled layers. Vales in red are p-values larger than 0.05. (EO: ELB oxic; WO: WLB oxic, WA: WLB anoxic; FO; FRX oxic; FA; FRX anoxic; ES: ELB shallow; ED: ELB deep; WS: WLB shallow; WD: WLB deep; FS: FRX shallow; FD: FRX deep)

| Pair | Data Type | R value | p (corrected) |
| --- | --- | --- | --- |
| EO-WO | Bacteria | 0.057 | 1.0000 |
| EO-WA | Bacteria | 0.954 | 0.0010 |
| EO-FO | Bacteria | 0.663 | 0.0010 |
| EO-FA | Bacteria | 0.960 | 0.0070 |
| WO-WA | Bacteria | 0.688 | 0.0040 |
| WO-FO | Bacteria | 0.553 | 0.0030 |
| WO-FA | Bacteria | 0.758 | 0.0150 |
| WA-FO | Bacteria | 1.000 | 0.0010 |
| WA-FA | Bacteria | 1.000 | 0.0170 |
| FO-FA | Bacteria | 1.000 | 0.0130 |
| ELB-WLB | Eukaryotes | 0.283 | 0.0015 |
| ELB-FRX | Eukaryotes | 0.971 | 0.0003 |
| WLB-FRX | Eukaryotes | 0.818 | 0.0003 |
| ES-ED | Eukaryotes | 0.570 | 0.0030 |
| ES-WS | Eukaryotes | 0.293 | 0.4620 |
| ES-WD | Eukaryotes | 0.857 | 0.0135 |
| ES-FS | Eukaryotes | 1.000 | 0.0420 |
| ES-FD | Eukaryotes | 1.000 | 0.0045 |
| ED-WS | Eukaryotes | 0.844 | 0.0030 |
| ED-WD | Eukaryotes | 0.554 | 0.0075 |
| ED-FS | Eukaryotes | 1.000 | 0.0015 |
| ED-FD | Eukaryotes | 1.000 | 0.0015 |
| WS-WD | Eukaryotes | 0.771 | 0.0105 |
| WS-FS | Eukaryotes | 0.946 | 0.0345 |
| WS-FD | Eukaryotes | 0.960 | 0.0060 |
| WD-FS | Eukaryotes | 1.000 | 0.0060 |
| WD-FD | Eukaryotes | 1.000 | 0.0030 |
| FS-FD | Eukaryotes | 0.870 | 0.0045 |

Table S3. Taxonomies of nodes identified in the network analysis (Figure 6).

| **Node Number** | **kingdom** | **class** | **family** | **genus** |
| --- | --- | --- | --- | --- |
| N001 | Bacteria | Actinobacteria | Sporichthyaceae | Candidatus Planktophila |
| N002 | Bacteria | Actinobacteria | Sporichthyaceae | hgcI clade |
| N003 | Bacteria | Actinobacteria | Sporichthyaceae | hgcI clade |
| N004 | Bacteria | Actinobacteria | Microbacteriaceae | Candidatus Aquiluna |
| N005 | Bacteria | Actinobacteria | Microbacteriaceae | Rathayibacter |
| N006 | Bacteria | Cytophagia | Cytophagia | Balneola |
| N007 | Bacteria | Flavobacteriia | Cryomorphaceae | Brumimicrobium |
| N008 | Bacteria | Flavobacteriia | Flavobacteriaceae | Polaribacter |
| N009 | Bacteria | Sphingobacteriia | Chitinophagaceae | uncultured |
| N010 | Bacteria | Sphingobacteriia | Sphingobacteriales | Pedobacter |
| N011 | Bacteria | Alphaproteobacteria | Rhodobacteraceae | uncultured |
| N012 | Bacteria | Alphaproteobacteria | Acetobacteraceae | Acidocella |
| N013 | Bacteria | Alphaproteobacteria | Rickettsiaceae | uncultured |
| N014 | Bacteria | Gammaproteobacteria | Gammaproteobacteria | none |
| N015 | Bacteria | Gammaproteobacteria | Piscirickettsiaceae | Marine Methylotrophic Group 3 |
| N016 | Bacteria | Gammaproteobacteria | Piscirickettsiaceae | uncultured |
| N017 | Bacteria | Opitutae | Puniceicoccaceae | uncultured |
| N018 | Bacteria | Verrucomicrobiae | Verrucomicrobia | uncultured |
| N019 | Eukaryota | Chloroplastida | Chlorophyceae | Mychonastes sp. NIES-2336 |
| N020 | Eukaryota | Chloroplastida | Chlorophyceae | uncultured Chlorophyta |
| N021 | Eukaryota | Chloroplastida | Trebouxiophyceae | Chlorellales |
| N022 | Eukaryota | Cryptomonadales | Cryptomonadales | none |
| N023 | Eukaryota | Prymnesiophyceae | Isochrysidaceae | none |
| N024 | Eukaryota | Prymnesiophyceae | Imantonia | uncultured marine picoeukaryote |
| N025 | Eukaryota | Holozoa | Acanthoecida | Acanthoecidae |
| N026 | Eukaryota | Holozoa | Acanthoecida | Acanthoecidae |
| N027 | Eukaryota | Holozoa | Craspedida | Codosigidae |
| N028 | Eukaryota | Alveolata | Intramacronucleata | Conthreep |
| N029 | Eukaryota | Alveolata | Dinophyceae | Peridiniphycidae |
| N030 | Eukaryota | Alveolata | SAR | none |
| N031 | Eukaryota | Stramenopiles | Chrysophyceae | Ochromonadales |
| N032 | Eukaryota | Stramenopiles | Chrysophyceae | Ochromonadales |
| N033 | Eukaryota | Stramenopiles | Eustigmatophyceae | Eustigmatales |
| N034 | Eukaryota | Stramenopiles | Eustigmatophyceae | Eustigmatales |
| N035 | Bacteria | Acidimicrobiia | Acidimicrobiaceae | CL500-29 marine group |
| N036 | Bacteria | Acidimicrobiia | Acidimicrobiaceae | CL500-29 marine group |
| N037 | Bacteria | Acidimicrobiia | Acidimicrobiales | Candidatus Microthrix |
| N038 | Bacteria | Acidimicrobiia | Actinobacteria | Acidimicrobiales |
| N039 | Bacteria | Actinobacteria | Cryptosporangiaceae | Fodinicola |
| N040 | Bacteria | Actinobacteria | Sporichthyaceae | Candidatus Planktophila |
| N041 | Bacteria | Actinobacteria | Sporichthyaceae | hgcI clade |
| N042 | Bacteria | Actinobacteria | Sporichthyaceae | hgcI clade |
| N043 | Bacteria | Actinobacteria | Sporichthyaceae | hgcI clade |
| N044 | Bacteria | Actinobacteria | Sporichthyaceae | hgcI clade |
| N045 | Bacteria | Actinobacteria | Microbacteriaceae | Agrococcus |
| N046 | Bacteria | Actinobacteria | Microbacteriaceae | Candidatus Aquiluna |
| N047 | Bacteria | Actinobacteria | Microbacteriaceae | Candidatus Aquiluna |
| N048 | Bacteria | Actinobacteria | Microbacteriaceae | Microbacteriaeae |
| N049 | Bacteria | Thermoleophilia | TM146 | uncultured actinobacterium |
| N050 | Bacteria | Cytophagia | Cyclobacteriaceae | Algoriphagus |
| N051 | Bacteria | Cytophagia | Cyclobacteriaceae | Algoriphagus |
| N052 | Bacteria | Cytophagia | Cytophagaceae | Hymenobacter |
| N053 | Bacteria | Cytophagia | Cytophagaceae | Cytophagales |
| N054 | Bacteria | Cytophagia | Flammeovirgaceae | Marivirga |
| N055 | Bacteria | Flavobacteriia | Cryomorphaceae | Fluviicola |
| N056 | Bacteria | Flavobacteriia | Cryomorphaceae | Fluviicola |
| N057 | Bacteria | Flavobacteriia | Cryomorphaceae | Fluviicola |
| N058 | Bacteria | Flavobacteriia | Cryomorphaceae | Fluviicola |
| N059 | Bacteria | Flavobacteriia | Cryomorphaceae | Fluviicola |
| N060 | Bacteria | Flavobacteriia | Cryomorphaceae | Cryomorphaceae |
| N061 | Bacteria | Flavobacteriia | Flavobacteriaceae | Arenibacter |
| N062 | Bacteria | Flavobacteriia | Flavobacteriaceae | Flavobacterium |
| N063 | Bacteria | Flavobacteriia | Flavobacteriaceae | Polaribacter |
| N064 | Bacteria | Flavobacteriia | Flavobacteriaceae | Sufflavibacter |
| N065 | Bacteria | Sphingobacteriia | Chitinophagaceae | Sediminibacterium |
| N066 | Bacteria | Sphingobacteriia | Chitinophagaceae | Sediminibacterium |
| N067 | Bacteria | Sphingobacteriia | Chitinophagaceae | uncultured |
| N068 | Bacteria | Sphingobacteriia | NS11-12 | uncultured bacterium |
| N069 | Bacteria | Sphingobacteriia | NS11-12 | Sphingobacteria |
| N070 | Bacteria | Sphingobacteriia | NS11-12 | uncultured bacterium |
| N071 | Bacteria | Sphingobacteriia | NS11-12 | Bacteroidetes |
| N072 | Bacteria | Sphingobacteriia | Sphingobacteriaceae | Pedobacter |
| N073 | Bacteria | Sphingobacteriia | Sphingobacteriaceae | Pedobacter |
| N074 | Bacteria | Chlorobia | Chlorobi_OPB56 | Chlorobi bacterium |
| N075 | Bacteria | Caldilineae | Caldilineaceae | Caldilineales |
| N076 | Bacteria | Gemmatimonadetes | Gemmatimonadaceae | Gemmatimonas |
| N077 | Bacteria | Phycisphaerae | Phycisphaeraceae | uncultured |
| N078 | Bacteria | Planctomycetacia | Planctomycetaceae | Blastopirellula |
| N079 | Bacteria | Planctomycetacia | Planctomycetaceae | Planctomyces |
| N080 | Bacteria | Planctomycetacia | Planctomycetaceae | Planctomyces |
| N081 | Bacteria | Planctomycetacia | Planctomycetaceae | Rhodopirellula |
| N082 | Bacteria | Alphaproteobacteria | Caulobacteraceae | Brevundimonas |
| N083 | Bacteria | Alphaproteobacteria | Caulobacteraceae | Phenylobacterium |
| N084 | Bacteria | Alphaproteobacteria | Hyphomonadaceae | Hyphomonas |
| N085 | Bacteria | Alphaproteobacteria | Rhodobacteraceae | Rhodobacter |
| N086 | Bacteria | Alphaproteobacteria | Rhodobacteraceae | uncultured |
| N087 | Bacteria | Alphaproteobacteria | Acetobacteraceae | Acidocella |
| N088 | Bacteria | Alphaproteobacteria | EF100-94H03 | uncultured bacterium |
| N089 | Bacteria | Alphaproteobacteria | Mitochondria | Nannochloropsis oceanica |
| N090 | Bacteria | Alphaproteobacteria | Rickettsiaceae | uncultured |
| N091 | Bacteria | Alphaproteobacteria | Chesapeake | uncultured bacterium |
| N092 | Bacteria | Alphaproteobacteria | SAR11 | SAR11 |
| N093 | Bacteria | Alphaproteobacteria | Erythrobacteraceae | Porphyrobacter |
| N094 | Bacteria | Alphaproteobacteria | Sphingomonadaceae | Novosphingobium |
| N095 | Bacteria | Alphaproteobacteria | Sphingomonadaceae | Novosphingobium |
| N096 | Bacteria | Alphaproteobacteria | Sphingomonadaceae | Novosphingobium |
| N097 | Bacteria | Alphaproteobacteria | Sphingomonadaceae | Sphingorhabdus |
| N098 | Bacteria | ARKICE-90 | ARKICE-90 | none |
| N099 | Bacteria | Betaproteobacteria | Alcaligenaceae | GKS98 freshwater group |
| N100 | Bacteria | Betaproteobacteria | Alcaligenaceae | GKS98 freshwater group |
| N101 | Bacteria | Betaproteobacteria | Comamonadaceae | BAL58 marine group |
| N102 | Bacteria | Betaproteobacteria | Comamonadaceae | Hydrogenophaga |
| N103 | Bacteria | Betaproteobacteria | Comamonadaceae | Hydrogenophaga |
| N104 | Bacteria | Betaproteobacteria | Comamonadaceae | Polaromonas |
| N105 | Bacteria | Betaproteobacteria | Comamonadaceae | uncultured |
| N106 | Bacteria | Betaproteobacteria | Oxalobacteraceae | Paucimonas |
| N107 | Bacteria | Betaproteobacteria | Betaproteobacteria | none |
| N108 | Bacteria | Deltaproteobacteria | Syntrophaceae | Smithella |
| N109 | Bacteria | Gammaproteobacteria | Halieaceae | OM60(NOR5) clade |
| N110 | Bacteria | Gammaproteobacteria | Legionellaceae | Legionella |
| N111 | Bacteria | Gammaproteobacteria | Oceanospirillaceae | Pseudospirillum |
| N112 | Bacteria | Gammaproteobacteria | Piscirickettsiaceae | Marine Methylotrophic Group 3 |
| N113 | Bacteria | Gammaproteobacteria | Xanthomonadaceae | Pseudoxanthomonas |
| N114 | Bacteria | OPB35 soil group | Verrucomicrobia | Verucomicrobia |
| N115 | Bacteria | Opitutae | Opitutaceae | Opitutus |
| N116 | Bacteria | Spartobacteria | Xiphinematobacteraceae | Candidatus Xiphinematobacter |
| N117 | Bacteria | Verrucomicrobiae | DEV007 | Verrucomicrobiaceae |
| N118 | Bacteria | Verrucomicrobiae | DEV007 | uncultured verrucomicrobium DEV019 |
| N119 | Bacteria | Verrucomicrobiae | Verrucomicrobiaceae | Prosthecobacter |
| N120 | Bacteria | Verrucomicrobiae | Verrucomicrobiaceae | Opitutus |
| N121 | Bacteria | Verrucomicrobiae | Verrucomicrobiaceae | Roseibacillus |
| N122 | Bacteria | Acidimicrobiia | Actinobacteria | uncultured bacterium |
| N123 | Bacteria | Actinobacteria | actinobacteria | none |
| N124 | Bacteria | Cytophagia | Cyclobacteriaceae | Algoriphagus |
| N125 | Bacteria | Flavobacteriia | Cryomorphaceae | Brumimicrobium |
| N126 | Bacteria | Flavobacteriia | Cryomorphaceae | Fluviicola |
| N127 | Bacteria | Flavobacteriia | Cryomorphaceae | uncultured |
| N128 | Bacteria | Flavobacteriia | Flavobacteriaceae | Ulvibacter |
| N129 | Bacteria | Sphingobacteriia | NS11-12 | uncultured Bacteroidetes bacterium |
| N130 | Bacteria | Sphingobacteriia | Sphingobacteriaceae | Pedobacter |
| N131 | Bacteria | Planctomycetacia | Planctomycetaceae | Planctomyces |
| N132 | Bacteria | Alphaproteobacteria | Hyphomonadaceae | Hyphomonas |
| N133 | Bacteria | Alphaproteobacteria | Rickettsiaceae | uncultured |
| N134 | Bacteria | Alphaproteobacteria | Sphingomonadaceae | Novosphingobium |
| N135 | Bacteria | Alphaproteobacteria | Sphingomonadaceae | Novosphingobium |
| N136 | Bacteria | Betaproteobacteria | Comamonadaceae | Polaromonas |
| N137 | Bacteria | Gammaproteobacteria | Legionellaceae | Legionella |
| N138 | Bacteria | Verrucomicrobiae | DEV007 | uncultured Verrucomicrobiaceae bacterium |
| N139 | Bacteria | Verrucomicrobiae | Verrucomicrobiaceae | uncultured |
| N140 | Bacteria | Actinobacteria | Sporichthyaceae | hgcI clade |
| N141 | Bacteria | Actinobacteria | Actinobacteria | none |
| N142 | Bacteria | Thermoleophilia | Thermoleophilia | uncultured bacterium |
| N143 | Bacteria | Flavobacteriia | Flavobacteriaceae | Flavobacterium |
| N144 | Bacteria | Sphingobacteriia | Chitinophagaceae | Sediminibacterium |
| N145 | Bacteria | Sphingobacteriia | Chitinophagaceae | Sediminibacterium |
| N146 | Bacteria | Sphingobacteriia | NS11-12 | uncultured bacterium |
| N147 | Bacteria | Chlorobia | OPB56 | uncultured bacterium |
| N148 | Bacteria | Planctomycetacia | Planctomycetaceae | uncultured |
| N149 | Bacteria | Alphaproteobacteria | Rhodospirillales | Reyranella |
| N150 | Bacteria | Alphaproteobacteria | Rickettsiaceae | uncultured |
| N151 | Bacteria | Betaproteobacteria | Alcaligenaceae | GKS98 freshwater group |
| N152 | Bacteria | Betaproteobacteria | Betaproteobacteria | none |
| N153 | Bacteria | Deltaproteobacteria | Syntrophaceae | Smithella |
| N154 | Eukaryota | Chloroplastida | Chlorophyceae | uncultured eukaryote |
| N155 | Eukaryota | Holozoa | Craspedida | Codosigidae |
| N156 | Eukaryota | Holozoa | Craspedida | Codosigidae |
| N157 | Eukaryota | Rhizaria | Clathrulinidae | Hedriocystis |
| N158 | Bacteria | Acidimicrobiia | Acidimicrobiaceae | CL500-29 marine group |
| N159 | Bacteria | Acidimicrobiia | Acidimicrobiaceae | CL500-29 marine group |
| N160 | Bacteria | Acidimicrobiia | Acidimicrobiales | Candidatus Microthrix |
| N161 | Bacteria | Acidimicrobiia | Acidimicrobiales | uncultured bacterium |
| N162 | Bacteria | Actinobacteria | Sporichthyaceae | Candidatus Planktophila |
| N163 | Bacteria | Actinobacteria | Sporichthyaceae | hgcI clade |
| N164 | Bacteria | Actinobacteria | Sporichthyaceae | hgcI clade |
| N165 | Bacteria | Actinobacteria | Sporichthyaceae | hgcI clade |
| N166 | Bacteria | Actinobacteria | Sporichthyaceae | hgcI clade |
| N167 | Bacteria | Actinobacteria | Microbacteriaceae | Candidatus Aquiluna |
| N168 | Bacteria | Actinobacteria | Microbacteriaceae | uncultured |
| N169 | Bacteria | Actinobacteria | Actinobacteria | none |
| N170 | Bacteria | Thermoleophilia | Gaiellales | uncultured bacterium |
| N171 | Bacteria | Thermoleophilia | Gaiellales | uncultured actinobacterium |
| N172 | Bacteria | Thermoleophilia | Thermoleophilia | uncultured bacterium |
| N173 | Bacteria | Thermoleophilia | TM146 | uncultured actinobacterium |
| N174 | Bacteria | Cytophagia | Cyclobacteriaceae | Algoriphagus |
| N175 | Bacteria | Flavobacteriia | Cryomorphaceae | Fluviicola |
| N176 | Bacteria | Flavobacteriia | Cryomorphaceae | uncultured |
| N177 | Bacteria | Flavobacteriia | Cryomorphaceae | Wandonia |
| N178 | Bacteria | Flavobacteriia | Flavobacteriaceae | Arenibacter |
| N179 | Bacteria | Flavobacteriia | Flavobacteriaceae | Flavobacterium |
| N180 | Bacteria | Flavobacteriia | Flavobacteriaceae | Flavobacterium |
| N181 | Bacteria | Flavobacteriia | Flavobacteriaceae | Flavobacterium |
| N182 | Bacteria | Flavobacteriia | Flavobacteriaceae | Flavobacterium |
| N183 | Bacteria | Flavobacteriia | Flavobacteriaceae | Flavobacterium |
| N184 | Bacteria | Flavobacteriia | Flavobacteriaceae | Flavobacterium |
| N185 | Bacteria | Flavobacteriia | Flavobacteriaceae | Flavobacterium |
| N186 | Bacteria | Flavobacteriia | Flavobacteriaceae | Flavobacterium |
| N187 | Bacteria | Flavobacteriia | Flavobacteriaceae | Flavobacterium |
| N188 | Bacteria | Flavobacteriia | Flavobacteriaceae | Flavobacterium |
| N189 | Bacteria | Flavobacteriia | Flavobacteriaceae | Sufflavibacter |
| N190 | Bacteria | Flavobacteriia | NS9 | uncultured bacterium |
| N191 | Bacteria | Flavobacteriia | Schleiferiaceae | Schleiferia |
| N192 | Bacteria | Sphingobacteriia | Chitinophagaceae | Sediminibacterium |
| N193 | Bacteria | Sphingobacteriia | Chitinophagaceae | Sediminibacterium |
| N194 | Bacteria | Sphingobacteriia | Chitinophagaceae | uncultured |
| N195 | Bacteria | Sphingobacteriia | Chitinophagaceae | uncultured |
| N196 | Bacteria | Sphingobacteriia | Chitinophagaceae | uncultured |
| N197 | Bacteria | Sphingobacteriia | Sphingobacteriales | uncultured bacterium |
| N198 | Bacteria | Sphingobacteriia | NS11-12 | uncultured bacterium |
| N199 | Bacteria | Sphingobacteriia | NS11-12 | uncultured bacterium |
| N200 | Bacteria | Sphingobacteriia | NS11-12 | uncultured bacterium |
| N201 | Bacteria | Sphingobacteriia | NS11-12 | uncultured Bacteroidetes bacterium |
| N202 | Bacteria | Sphingobacteriia | PHOS-HE51 | uncultured bacterium |
| N203 | Bacteria | Sphingobacteriia | Sphingobacteriaceae | Pedobacter |
| N204 | Bacteria | uncultured bacterium | SR1 | none |
| N205 | Bacteria | Chlorobia | OPB56 | uncultured bacterium |
| N206 | Bacteria | Caldilineae | Caldilineaceae | uncultured |
| N207 | Bacteria | Bacilli | Paenibacillaceae | Paenibacillus |
| N208 | Bacteria | Phycisphaerae | Phycisphaeraceae | CL500-3 |
| N209 | Bacteria | Planctomycetacia | Planctomycetaceae | Pirellula |
| N210 | Bacteria | Planctomycetacia | Planctomycetaceae | Planctomyces |
| N211 | Bacteria | Planctomycetacia | Planctomycetaceae | uncultured |
| N212 | Bacteria | Alphaproteobacteria | Rhodobacteraceae | Rhodobacter |
| N213 | Bacteria | Alphaproteobacteria | Rhodobacteraceae | Rhodobacter |
| N214 | Bacteria | Alphaproteobacteria | Rhodospirillales | Reyranella |
| N215 | Bacteria | Alphaproteobacteria | Rickettsiaceae | uncultured |
| N216 | Bacteria | Alphaproteobacteria | Sphingomonadaceae | Novosphingobium |
| N217 | Bacteria | Betaproteobacteria | Alcaligenaceae | GKS98 freshwater group |
| N218 | Bacteria | Betaproteobacteria | Alcaligenaceae | GKS98 freshwater group |
| N219 | Bacteria | Betaproteobacteria | Comamonadaceae | Hydrogenophaga |
| N220 | Bacteria | Betaproteobacteria | Comamonadaceae | Polaromonas |
| N221 | Bacteria | Betaproteobacteria | Oxalobacteraceae | Paucimonas |
| N222 | Bacteria | Betaproteobacteria | Methylophilaceae | Methylotenera |
| N223 | Bacteria | Betaproteobacteria | Betaproteobacteria | none |
| N224 | Bacteria | Deltaproteobacteria | Bacteriovoracaceae | Peredibacter |
| N225 | Bacteria | Deltaproteobacteria | Syntrophaceae | Smithella |
| N226 | Bacteria | Gammaproteobacteria | Oceanospirillaceae | Pseudospirillum |
| N227 | Bacteria | Gammaproteobacteria | Pseudomonadaceae | Pseudomonas |
| N228 | Bacteria | Opitutae | Opitutaceae | Opitutus |
| N229 | Bacteria | Verrucomicrobiae | Verrucomicrobiaceae | Prosthecobacter |
| N230 | Bacteria | uncultured bacterium | WCHB1-60 | none |
| N231 | Eukaryota | Chloroplastida | Chlorophyceae | uncultured eukaryote |
| N232 | Eukaryota | Holozoa | Craspedida | Codosigidae |
| N233 | Eukaryota | Holozoa | Craspedida | Codosigidae |
| N234 | Eukaryota | Rhizaria | Clathrulinidae | Hedriocystis |
